# Severe obesity increases risk of graft loss in pediatric heart transplantation

**DOI:** 10.1016/j.xjon.2025.04.023

**Published:** 2025-05-19

**Authors:** Joshua D. Sparks, Sarah J. Wilkens, Andrea Nicole Lambert, Deborah Kozik, Jaimin R. Trivedi, Bahaaldin Alsoufi

**Affiliations:** a Department of Pediatrics, University of Louisville and Norton Children's Hospital, Louisville, Ky; b Department of Cardiothoracic Surgery, University of Louisville and Norton Children's Hospital, Louisville, Ky

**Keywords:** heart transplantation, pediatrics, survival, obesity, severe obesity, heart failure

## Abstract

**Objectives:**

Severe obesity is an established risk factor for adverse cardiovascular events and heart transplantation (HT) outcomes in adults. However, the effect of severe obesity on children after HT is not well studied. We aimed to examine the prevalence and effect of severe obesity on pediatric HT.

**Methods:**

We evaluated children (>8 years) listed for HT using the United Network for Organ Sharing database. Severe obesity was defined per Centers for Disease Control and Prevention criteria using body mass index. Our study comprised 2 groups: a severe obesity group (n = 212, 8%) and a control group (n = 2417, 92%) consisting of the remaining children. We compared characteristics and outcomes between the 2 groups.

**Results:**

After listing, there was no difference in transplant rate or waitlist mortality between the severe obesity and control groups (*P* = .89). Children with severe obesity were less likely to have congenital heart disease and more likely to be Black, have greater levels of creatinine, be supported with a left ventricular assist device, and receive grafts from older donors. Waitlist duration was comparable (*P* = .23). Incidences of primary graft dysfunction (*P* = .91), stroke (*P* = .36), dialysis (*P* = .18), and acute rejection (*P* = .4) were similar. However, severe obesity group had significant survival disadvantage (10 years: 47% vs 64%, *P* = .01), particularly in children older than 11 years, with diverging outcomes starting around 4 years posttransplant in those older than 15 years. Cox regression identified severe obesity as independent mortality risk factor (hazard ratio, 1.88; *P* = .0003), along with age, gender, race, congenital heart disease, creatinine, extracorporeal membrane oxygenation, and donor age.

**Conclusions:**

There is a pressing need to improve assessment and treatment of obesity in children with end-stage heart failure awaiting transplantation. Although early survival rates are comparable, med- and long-term outcomes are concerning for severely obese children after heart transplant. Though unclear, the pathophysiologic effects are likely due to accelerated allograft vasculopathy from the metabolic derangement of obesity. Particularly in older children and adolescents, severe obesity should be considered a modifiable risk factor and aggressively managed before and after transplantation.

## Graphical abstract

**Figure:**
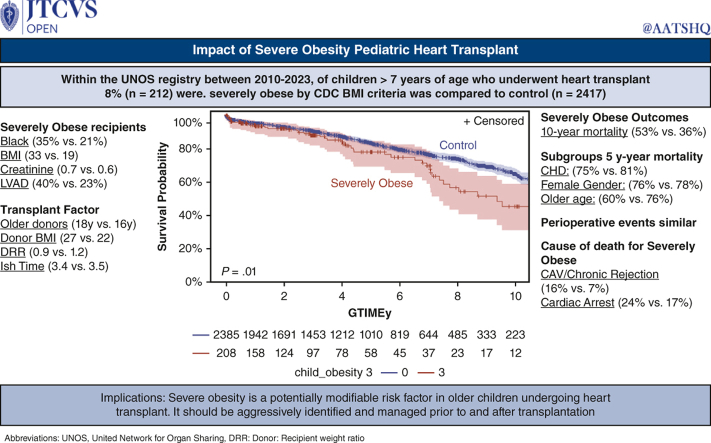

**Figure:**
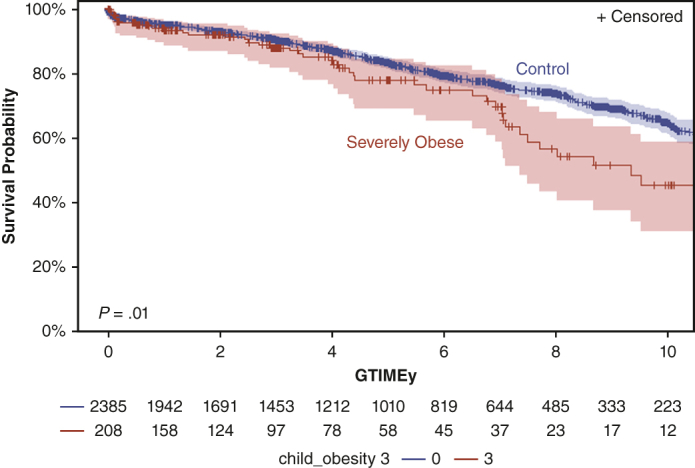
Worse heart transplant survival in children with severe obesity.

**Central Message**

Severe obesity at transplant reduces long term survival after pediatric heart transplantation.

**Perspective**

Severe obesity may be a significant risk factor for outcomes after heart transplant in children. The UNOS database allowed for a retrospective analysis of children older than 8 years of age with severe obesity and its impact on transplant outcomes.

Obesity is a known epidemic among adults and children in the United States. The National Health and Nutrition Examination Survey conducted in 2018 demonstrated that 31%, 42%, and 9% of adults have overweight, obesity, and severe obesity, respectively. In adults, the deleterious effects of obesity on the cardiovascular system are well described and include systemic hypertension, type 2 diabetes mellitus, dyslipidemia, sleep apnea, and pulmonary hypertension.^1^ These consequences have the potential to have deleterious effects after heart transplantation (HT).

These concerns have been supported by a contemporary review of the United Network for Organ Sharing (UNOS) database in adult listed patients which demonstrated that body mass index (BMI) had a significant impact on all phases of transplantation, including the likelihood of being transplanted to a greater risk of death after transplant.^2^ As a result, many adult centers consider a BMI greater than 35 kg/m^2^ to be a contraindication.^3^ This may, however lead to selection bias in outcome assessments after HT.

Unfortunately, the National Health and Nutrition Examination Survey study revealed a concerning trend in children aged 2 to 19 years, who had a prevalence of 16%, 19%, and 6% for overweight, obese, and severe obesity, respectively. These findings cause a significant ethical dilemma for pediatric HT because, despite what has been shown in the adult population, the comorbidities in the adult population are inconsistently found in children presumably because they are a consequence of obese physiology that has not yet had time to develop. Currently, there are insufficient data to support a specific BMI cutoff for pediatric HT,^4^ and there is limited information regarding its effect on outcome. There may be disparate selection bias or epidemiologic differences in obese children with heart failure, and it is unclear whether obesity is an independent risk factor. It challenges proper organ stewardship whether severe obesity should preclude HT. We conducted a contemporary analysis of the UNOS database to evaluate the prevalence and impact of severe obesity on outcomes in children awaiting HT.

## Methods

**Figure 1:**
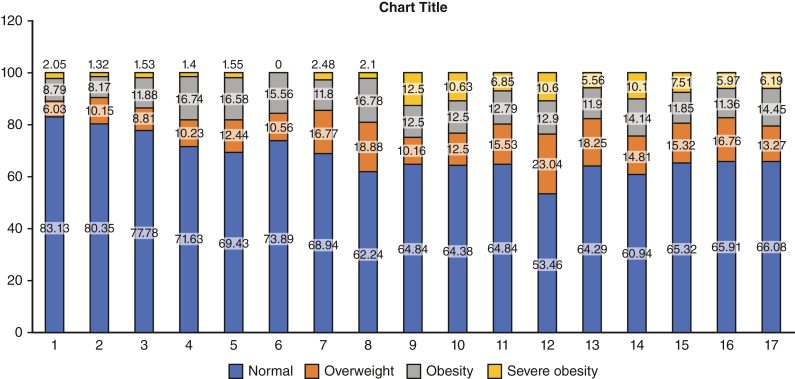
The frequency analysis of overweight, obesity, and severe obesity in children from the UNOS database who underwent heart transplantation from January 1, 2010, to December 30, 2023. *UNOS*, United Network for Organ Sharing.

The UNOS database was queried for all pediatric recipients of HT (<18 years of age) from January 1, 2010, to December 30, 2023. The population was separated by degree of obesity as defined by the Centers for Disease Control and Prevention using currently accepted normal values as described herein. Figure 1 demonstrates the cohort studied. In total, 5794 children underwent HT. The incidence of severe obesity was negligible for children younger than the age of 8 years (<1.5%); therefore, the primary analysis was restricted to recipients older than the age of 8 years (n = 2629).

Recipient characteristics and pretransplant variables were recorded including age, race, gender , weight, height, diagnoses, listing status, creatinine, total bilirubin, duration on the wait list, and heart failure support while waiting. Perioperative data included ischemic time and postoperative mechanical circulatory support. Donor demographics, cause of death, and downtime were recorded. The primary end point was death after transplantation. Secondary end points include perioperative complications such as primary graft failure, rejection, and noncardiac organ failure.

The database provides BMI data at the time of transplant. The Centers for Disease Control and Prevention classifies child obesity as follows: underweight (BMI <fifth percentile), normal weight (BMI fifth-85th percentile), overweight (BMI >85th but <95th percentile), obese (BMI >95th percentile), and severely obese (BMI >120% of the 95th percentile or an absolute value of 35 kg/m^2^). For our analysis, we initially categorized the cohort into 4 groups: normal (<85th percentile), overweight (85th-95th percentile), obese (>95th percentile), and severely obese (>120% of the 95th percentile). Given that outcomes for severely obese group were worse and the other groups all have otherwise comparable outcomes, we separated our patients into a severe obesity group (>120% of the 95th percentile) and a control group, the combined children from the other weight categories. We conducted group comparisons regarding primary and secondary end points. This study received approval from our institutional review board (no. 14.0343 approved on August 17, 2022) with a waiver of individual consent.

### Statistical Analysis

The data were initially analyzed using descriptive statistics and reported using median (interquartile range, or IQR) for continuous variables and % (n) for categorical variables. The differences between the study groups were evaluated using univariate statistics. Nonparametric Kruskal-Wallis test was used for continuous variable and χ^2^ test was used for categorical variables. Kaplan-Meier survival estimates evaluated posttransplant survival and the log-rank test compared between the study groups. A Cox-proportional hazard model was generated and variables with *P* value < .15 were on table were included in the model. In addition, gender was added to the model. The severe obesity variable was used in the cox model to evaluate its impact on long-term survival outcome. All the analysis was conducted using SAS 9.4 software (SAS Institute) at 95% confidence interval.

## Results

Overall, the median age was 14 years (IQR, 11-16), and there were 1085 (41%) female patients, 1275 patients of (48%) non-White race, 913 (34%) diagnosed with congenital heart disease, and 630 (24%) supported with mechanical circulatory support. The median BMI for the entire cohort was 20 (17-24) kg/m2.

Table 1 compares the severe obesity group (n = 212, median BMI of 33 [31-36]) and the control group as defined previously (n = 2417, median BMI 13 [17-23]). Patients in the severe obesity group were more likely to be of Black race (35% vs 21%, *P* < .01) but of similar age and gender. At the time of transplant, they had a greater creatinine levels (0.6 [0.5-0.8] vs 0.7 [0.5-0.9], *P* < .01), were less likely to have congenital heart disease (36% vs 18%, *P* < .01), and more likely to be on ventricular assist device (VAD) support (40% vs 23%, *P* < .01). There were no differences in total bilirubin, mechanical ventilation, extracorporeal membrane oxygenation (ECMO) support, or time on the waitlist. They had a lower ischemic time but both were less than 4 hours (3.4 [2.9-3.9] hours vs 3.5 [3.0-4.1] hours, *P* = .03).

**Table 1:** Group comparison

Transplant variable	Control (n = 2417)	Severe obesity (n = 212)	*P* Value
Age, y	14 (11-16)	13 (11-15)	.11
Gender (female)	41%	41%	.82
Race			
Black	21%	35%	<.01
White	53%	39%	
Other	26%	26%	
BMI, kg/m^2^	19 (17-23)	33 (31-36)	<.01
Creatinine, mg/dL	0.6 (0.5-0.8)	0.7 (0.5-0.9)	<.01
Bilirubin, mg/dL	0.7 (0.4-1.2)	0.6 (0.4-1.0)	.07
Diagnosis			
CHD	36%	18%	<.01
NICM	21%	41%	
Other	43%	41%	
Mechanical ventilation	5%	6%	.73
ECMO	3%	3%	.87
LVAD	23%	40%	<.01
Wait time∗	50 (15-150)	48 (12-102)	.23
Ischemia time	3.5 (3.0-4.1)	3.4 (2.9-3.9)	.03
Donor age, y	16 (13-21)	18 (16-23)	<.01
Donor BMI	22 (20-26)	27 (24-30)	<.01
Donor COD			
Anoxia	33%	24%	.07
CVA	7%	7%	
Head trauma	57%	66%	
Other	3%	3%	
Donor-recipient weight ratio	1.2 (1.1-1.4)	0.9 (0.8-1.1)	<.01

*BMI*, Body mass index; *CHD*, congenital heart disease; *NICM*, nonischemic cardiomyopathy; *ECMO*, extracorporeal membrane oxygenation; *LVAD*, left ventricular assist device, *COD*, cause of death; *CVA*, cerebrovascular accident/stroke.

∗ Includes inactive time.

They also had older donors (18 years [16-23] vs 16 years [13-21], *P* < .01) with a greater donor BMI (27 [24-30] vs 22 [20-26], *P* < .01) and a lower donor/recipient weight ratio (0.9 [0.8-1.1] vs 1.2 [1.1-1.4], *P* < .01). There were no differences in donor causes of death.

There was no difference between the severe obesity and control groups with respect to transplant wait-time (*P* = .23), incidence of primary graft dysfunction (*P* = .91), postoperative stroke (*P* = .36), need for postoperative dialysis (*P* = .18), acute rejection (*P* = .4), or death (*P* = .96).

**Figure 2:**
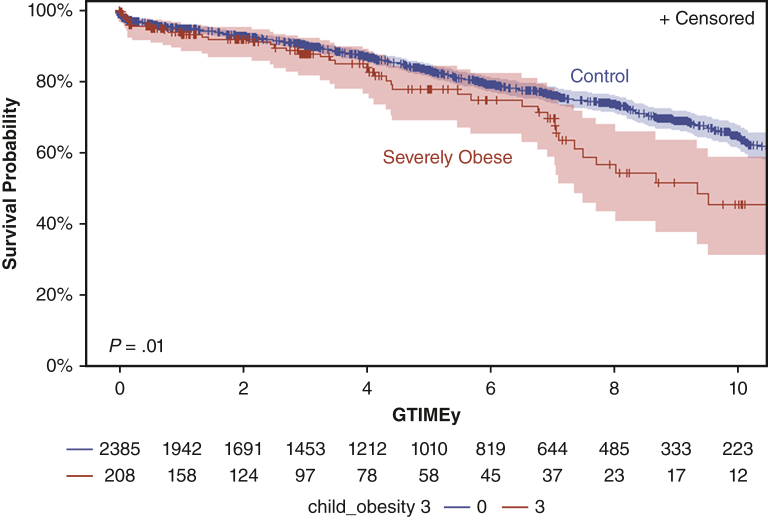
Reduced survival after pediatric heart transplantation (age 8-18 years) from the UNOS dataset severe obesity versus control (those without severe obesity) at 10-year (45%; 95% CI. 32%-57% vs 64%; 95% CI, 61%-67%, *P* = .01). *UNOS*, United Network for Organ Sharing.

**Figure 3:**
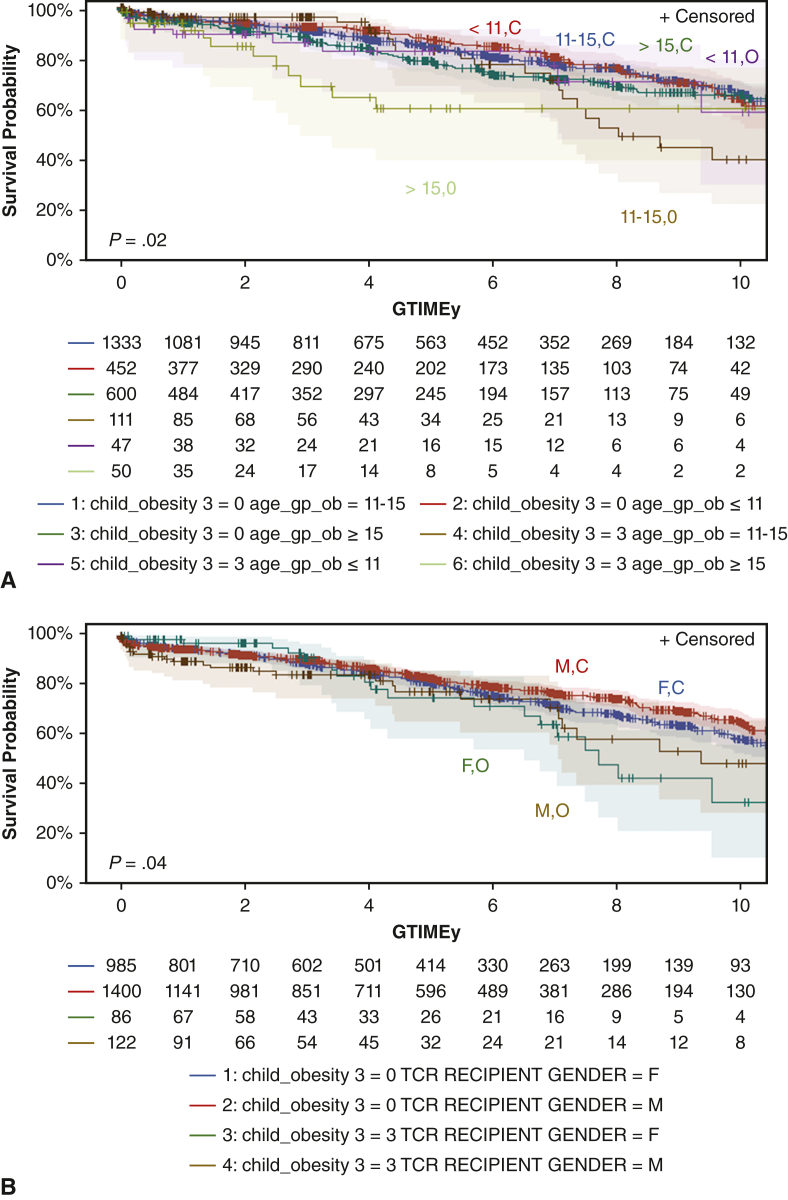

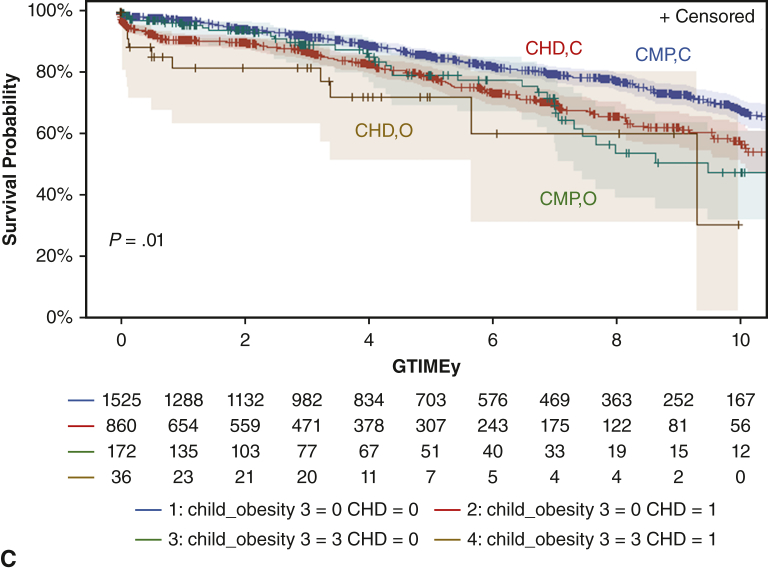
Reduced 5-year survival in heart transplant recipients with obesity when with regards to age (A), gender (B), and diagnosis (C). A, Comparison of severely obese (O) versus control (C) broken down by age groups of those younger than 11 years of age (<11) (82%; 95% CI, 71%-92% vs 87%; 95% CI, 85%-89%), aged 11 to 15 years (11-15) (82%; 95% CI, 73% - 91%) vs 84%; 95% CI, 82%-86%), and older than 15 years of age (>15) (59%; 95% CI, 41%-77%) versus 78%; 95% CI, 74%-82%), *P* = .02. B, Comparison of severely obese male (M) (79%; 95% CI, 70%-88% vs 84%; 95% CI, 82%-86%) and female (F) (77%; 95% CI, 66%-88% vs 82%; 95% CI, 81%-84%), *P* = .04. C, Comparison of those with severe obesity and congenital heart disease (CHD) (72%; 95% CI, 57%-87% vs 79%; 95% CI, 77%-81%) compared with cardiomyopathy (CMP) (79%; 95% CI, 71%-87% vs 85%; 95% CI, 83%-87%), *P* = .01. *CI*, Confidence interval.

**Figure E1:**
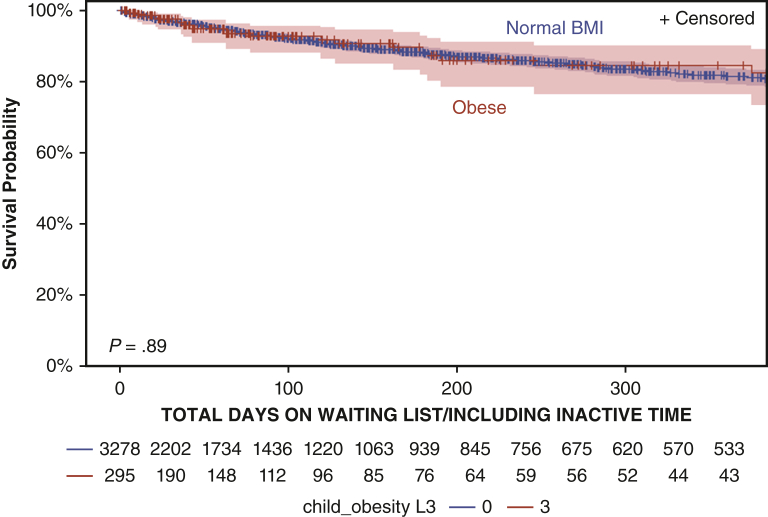
Waitlist survival for severely obese children versus control (88%; 95% CI, 85%-91% vs 88%; 95% CI, 87%-89%), *P* = .89. *CI*, Confidence interval; *BMI*, body mass index.

**Figure E2:**
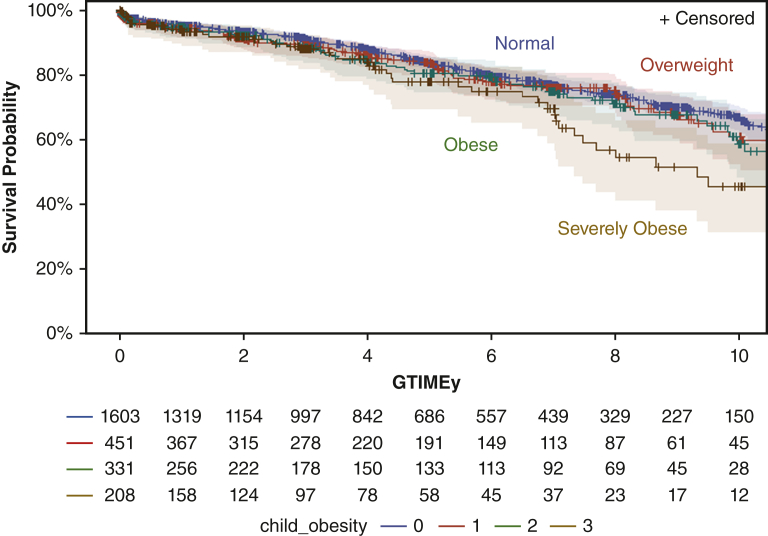
Survival breakdown on the basis of severity of Centers for Disease Control and Prevention classification of body mass index: normal [83%; 95% CI, 82% - 84%)], overweight (83%; 95% CI, 80%-86%), obese (80%; 95% CI, 76%-84%), and severely obese (78%; 95% CI, 72%-84%). *CI*, Confidence interval.

**Figure E3:**
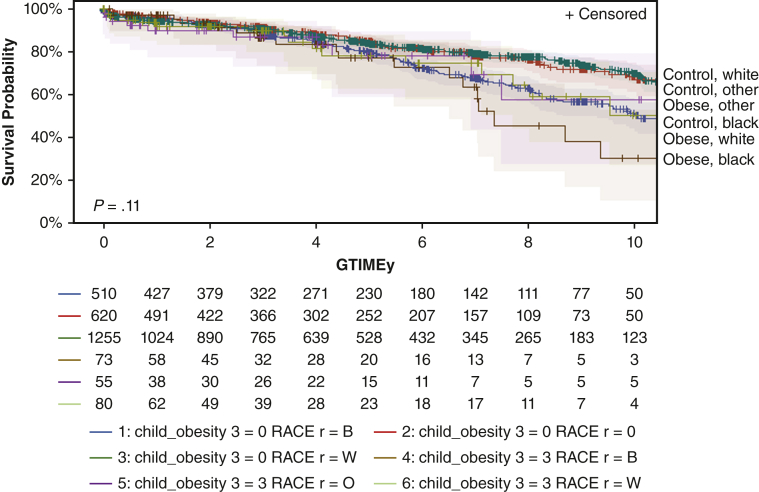
Heat transplant survival for severely obese children versus control with regards to race: *White* (W) (78%; 95% CI, 67%-89% vs 84%; 95% CI, 82%-86%), *Black* (B) (77%; 95% CI, 66%-88% vs 79%; 95% CI, 75%-83%), and other (O) (78%; 95% CI, 65%-91% vs 85%; 95% CI, 83%-87%), *P* = .11. *CI*, Confidence interval.

Figure 2 demonstrates the survival analysis of death after transplant, demonstrating worse survival for patients with severe obesity as opposed to those the control group, with increased attrition at approximately 4 years after transplant, and by 10 years post-HT, more than one half of the patients in the severely obese group had died (53% vs 36%, *P* = .01). There was no difference in waitlist mortality (Figure E1). Survival on the basis of varying degrees of obesity can be found in Figure E2. This survival disadvantage persisted when subgrouped by age, gender, and cardiac diagnosis (Figure 3, *A*-*C*). In fact, it was more pronounced in children with a diagnosis of congenital heart disease (75% vs 81% at 5 years, *P* < .01), older age (60% vs 76% at 5 years, *P* < .01), and female gender (77% vs 79% at 5 years, *P* = .04), Race appeared to separate survival but failed to reach statistical significance (Figure E3).

On multivariate analysis in Table 2, severe obesity remained an independent risk factor for death or graft loss (hazard ratio [HR], 1.88; *P* = .0003) second only to ECMO (HR, 1.9; *P* = .009). Other risk factors included BMI >95th percentile (HR, 1.38; *P* = .026), age (HR, 1.07; *P* = .0013), female gender (HR, 1.26 95%; *P* = .011), Black race (HR, 1.75; *P* ≤ .0001), White race (HR, 0.56; *P* ≤ .001), creatinine (HR, 1.07; *P* = .019), total bilirubin (HR, 1.08, *P* < .01), and donor age (HR, 1.02; *P* = .009).

**Table 2:** Cox regression

Variable	Hazard	*P* value
ECMO at transplant	1.901	.009
Severely obese BMI, kg/m^2^	1.875	.0003
Black race	1.752	<.0001
Obese BMI, kg/m^2^	1.376	.0262
Overweight BMI, kg/m^2^	1.276	.053
Female gender	1.246	.011
Total bilirubin	1.081	<.0001
Recipient age	1.065	.0013
Serum creatinine at time of transplant	1.065	.0189
Donor age, y	1.019	.0018
Donor-recipient weight ratio	1.018	.9239
Ischemic time, h	1.004	.9325
Days waiting∗	1	.43709
Donor BMI, kg/m^2^	0.979	.0599
LVAD	0.892	.3622
Ventilator at Transplant	0.952	.805
Cardiomyopathy vs CHD	0.577	<.0001
other vs CHD	0.528	<.0001

*ECMO*, Extracorporeal membrane oxygenation; *BMI*, body mass index; *LVAD*, left ventricular assist device; *CHD*, congenital heart disease.

∗ Includes inactive time.

There was no difference in perioperative events including primary graft failure, acute rejection, stroke, or need for postoperative dialysis. The cause of death was significantly different for those with severe obesity as compared with normal weight. Severely obese recipients were significantly more likely to die from chronic rejection, likely representing coronary artery vasculopathy (16% vs 7%) and cardiac arrest (24% vs 17%) (Table 3). When we evaluated BMI as a continuous variable, the hazard was 1.04, *P* = .0007 (full table in Table E1).

**Table 3:** Perioperative outcomes and recipient cause of death

Outcome events	Normal BMI	Severe obesity	*P* value
	n = 2417	n = 212	
Perioperative events			
PGD	1.30%	1.40%	NS
Acute rejection	15%	12%	
Stroke	3%	1.40%	
Dialysis	8%	11%	


Recipient cause of death	n = 383	n = 38	
Acute rejection	9%	5%	<.01
Chronic rejection	7%	16%	
Cardiac arrest	17%	24%	
Multiorgan failure	8%	16%	
Other	59%	39%	

*BMI*, Body mass index; *PGD*, primary graft dysfunction; *NS*, not significant.

## Discussion

Obesity poses a significant challenge in managing children with end-stage heart failure awaiting transplantation. These patients often struggle with weight loss as the result of cardiometabolic limitations such as cachexia and muscle loss.^5^^,^^6^ Our study indicates that severe obesity negatively impacts mid- and long-term survival posttransplant. With waitlist and perioperative survival rates being similar, the risks associated with severe obesity appear to stem from cumulative complications over time rather than preoperative, surgical, or donor factors.

Although the relationship between obesity and graft loss remains unclear, we speculate that it may contribute to known comorbidities associated with cardiac allograft vasculopathy (CAV). CAV is a nearly ubiquitous complication that develops late after HT but earlier in older recipients, affecting about one half of adolescent recipients by 10 years after transplant and being the leading cause of graft loss.^5^^,^^6^ In our study, mortality was described as chronic rejection and cardiac arrest, which we suspect are surrogates for CAV.

Our data revealed a significant late mortality hazard associated with severe obesity. In addition, although the timing of the hazards differ, it was comparable with that of ECMO support, a well-known risk factor for transplant outcome. Although early outcomes were similar between groups, after approximately 4 years, the mortality increased in the severely obese group, with more than 50% of the severe obesity group having experienced graft loss by 8 years post transplant. This risk appeared to be exacerbated by other known risk factors like older age at transplant, Black race, CHD diagnosis, and female gender.

With respect to age, specifically, regardless of the age at time of transplant, it was interesting to note that the mortality for the severely obesity group appeared to worsen around 11 years of age regardless of the age at which they were transplanted, suggesting that the survival disadvantages occurred in teenagers approaching early adulthood. Both older recipient and donor ages have been linked to increased CAV risk,^7^ coinciding with critical psychosocial and physiological changes, including risk-taking behaviors and noncompliance.^8^^,^^9^ It is possible, however, that the risk is not merely behavioral. The impact of puberty on cardiometabolic physiology is significant. Changes in sex hormones, growth, insulin sensitivity, and inflammatory responses affect cardiovascular disease development in obese children.^10^ Elevated circulating free fatty acids increase oxidative stress, reducing ATP availability and potentially impacting allograft function and longevity.^10^, ^11^, ^12^ It can be reasonably speculated that these changes in metabolism associated with becoming an adult may increase the risk of hyperglycemia, diabetes, and ultimately coronary artery disease/CAV.

Further complicating what has already been described previously, obesity also raises the risk of diabetes posttransplant, particularly among already high-risk groups (eg, Black race, older age, greater BMI). It activates proinflammatory cytokines, leading to inflammation, further oxidative stress, and endothelial dysfunction, contributing to cardiovascular disease. Although incompletely researched, the emerging concept of obesity as an inflammatory disease might increase the risk of chronic rejection, the development of donor antibodies, and ultimately early graft loss. These issues are further compounded by medications like tacrolimus and corticosteroids, which are diabetogenic and lead to chronic hypertension, dyslipidemia, and kidney disease.^10^, ^11^, ^12^

A common challenge when caring for a listed patient with obesity is assessing proper donor size or the potential of an obese donor being greater risk, such as older age, a greater BMI, and lower donor-to-recipient weight ratios. Although our donor group had a greater BMI (27 vs 22) and a lower weight ratio (0.9 vs 1.2), these factors did not independently affect outcome.

Our study raises the question of whether transplantation should be delayed for intervention Possible strategies to consider in select cases could include bariatric surgery with or without the use of a VAD as a “bridge to weight loss,” and/or employing new weight loss medications like incretin hormone receptor agonists (gastric inhibitory polypeptide and glucagon-like peptide-1).

The rationale for using VADs solely for weight loss in stable patients remains unclear, especially in children, with reports of limited success and greater complication rates.^13^^,^^14^ However, recently emerging data in adult patients show some promise; recent studies indicate that bariatric surgery during VAD support can lead to weight reduction and improved transplantation outcomes.^15^ A Cochranereview found bariatric surgery performed concurrently with left VAD to be a safe bridge to transplantation.^16^ In addition, the American Academy of Pediatrics now recommends consideration of weight-loss surgery as part of treatment for obesity. All this said, caution should be exercised when applying these concepts to children with heart failure. Surgical risks are likely greater and the risk profile of applying adult VAD devices to children are different. This may have a strong impact of the pro therapeutic index for these procedures. Further study is needed in this area.

More importantly, this study raises broader questions about obesity in the pediatric transplant recipient. Obesity appears to carry an ongoing risk after transplant and the cumulative risk may be greater than the perioperative time period. In fact, our study suggests that the risk is greater as the recipient approaches adolescence, although very young children were not assessed in our study. Recent advances in the medical management of obesity, particularly, with the use of glucagon-like peptide-1 agonists, is showing promise. Consideration of these types of intervention after transplant may carry a heavier importance than during the perioperative period.

## Limitations

This study has several limitations inherent to retrospective database analyses, including potential confounding variables, data entry inconsistencies, selection and recall bias, limited granularity, and reduced generalizability. In addition, patients younger than 8 years of age were excluded, although their low incidence of obesity is unlikely to have significantly impacted outcomes. Infants and young children with severe obesity may present confounding factors related to genetic and metabolic disorders, such as Bardet-Biedl syndrome, which could introduce different physiological substrates affecting the results. Furthermore, severe obesity may serve as a surrogate marker for posttransplant diabetes, which could not be statistically assessed as an independent hazard in this study's design.

Lastly, a momentary BMI, which is so largely determined on the basis of weight in a patient who may have fluid shifts at times including large percentages of his/her body weight could lead to a miscalculation of true BMI on the basis of nonfluid body mass. This is why we believed using BMI as a continuous variable may hold less utility than as categorical.

## Conclusions

There is a pressing need to better assess and treat obesity in children in need of HT. Although early survival rates are similar, the mid- and long-term outcomes are concerning. Although unclear, the pathophysiologic effects are likely due to accelerated allograft vasculopathy spurred on by the metabolic derangement of obesity. Particularly in older children and adolescents, severe obesity should be considered a modifiable risk factor and aggressively managed before and after transplantation.

## Conflict of Interest Statement

The authors reported no conflicts of interest.

The *Journal* policy requires editors and reviewers to disclose conflicts of interest and to decline handling or reviewing manuscripts for which they may have a conflict of interest. The editors and reviewers of this article have no conflicts of interest.

## Figures and Tables

**Table E1:** Cox proportional hazard for mortality using BMI as continuous variable

Parameter	HR	*P* value
ECMO at transplant	1.837	.0123
Black race	1.694	<.0001
Female gender	1.269	.0112
Donor recipient weight ratio	1.135	.5247
Total bilirubin mg/dL	1.083	<.0001
Serum creatinine at time of transplant	1.067	.0184
Recipient age	1.042	.0503
Recipient BMI, kg/m^2^	1.042	.0007
Donor age, y	1.018	.0172
Days waiting∗	1	.4425
Ischemic time, h	0.998	.9642
Other race	0.974	.8287
Donor BMI, kg/m^2^	0.97	.0253
LVAD	0.883	.3246
Ventilator at transplant	0.851	.4184
Cardiomyopathy vs CHD	0.568	<.0001
Other vs CHD	0.524	<.0001

*HR*, Hazard ratio; *ECMO*, extracorporeal membrane oxygenation; *BMI*, body mass index; *LVAD*, left ventricular assist device; *CHD*, congestive heart disease.

∗ Includes inactive time.
